# Impact of Childhood Trauma on Suicidal Behavior in Patients with Schizophrenia and Schizoaffective Disorder

**DOI:** 10.1192/j.eurpsy.2026.12186

**Published:** 2026-07-31

**Authors:** R. Chakchouk, E. Maaloul, S. Ellouze, Y. Kammoun, M. Turki, J. Aloulou

**Affiliations:** Psychiatry B Department, CHU Hedi Chaker Sfax, Sfax, Tunisia

## Abstract

**Introduction:**

Childhood trauma is a major risk factor influencing the clinical trajectory of schizophrenia spectrum disorders. Its association with suicidal behavior underscores the need for early recognition of trauma-related vulnerabilities to enhance suicide prevention in patients with schizophrenia and schizoaffective disorder.

**Objectives:**

This work aims to assess the patterns of childhood trauma and suicidal behavior among patients with schizophrenia and schizoaffective disorder.

**Methods:**

A cross-sectional descriptive study was conducted among 31 outpatients diagnosed with schizophrenia or schizoaffective disorder. Childhood trauma was evaluated using the Childhood Trauma Questionnaire – Short Form (CTQ-SF), and suicidality was assessed through structured clinical questions addressing lifetime and current suicidal ideation and attempts.

**Results:**

Overall, 77.4% of patients reported at least one form of maltreatment. The mean CTQ total score was 56, indicating moderate exposure to childhood trauma across the sample. The most frequent trauma subtypes were emotional abuse (13.5 ± 5.2) andemotional neglect (13.4 ± 4.3), followed by physical abuse (11.4 ± 5.5), physical neglect (9.7 ± 3.4), and sexual abuse (8.0 ± 5.5). Severe forms were most frequent for emotional (38.7%) and physical abuse (38.7%). Overall, 77.4% of participants reported at least one form of maltreatment.

Regarding suicidality, 51.6% of participants reported lifetime suicidal ideation, and 16% had a history of suicide attempts.

The CTQ total score was significantly higher in patients with a history of suicide attempts.

**Conclusions:**

Childhood trauma appears to play a crucial role in the emergence of suicidal behavior among patients with schizophrenia and schizoaffective disorder. These findings highlight the need for systematic assessment of early adverse experiences in clinical practice to improve suicide prevention and patient care.

**Disclosure of Interest:**

None Declared

